# Navigating Between the Plots: A Narratological and Ethical Analysis of Business-Related Conspiracy Theories (BrCTs)

**DOI:** 10.1007/s10551-020-04612-3

**Published:** 2020-09-14

**Authors:** Mathieu Alemany Oliver

**Affiliations:** Social & Innovation Marketing Lab, TBS Business School, 1 Place Alfonse Jourdain - CS 66810, 31068 Toulouse Cedex 7, France

**Keywords:** Business-related conspiracy theory (BrCT), Structural narratology, Critical CSR, Political CSR

## Abstract

This paper introduces the concept of business-related conspiracy theories (BrCTs). Drawing on Aristotelian virtue ethics and undertaking a narratological and ethical analysis of 28 BrCTs found online, I emphasize that BrCTs are narratives with structures rooted in other latent macro- and meta-narratives, including centuries-old myths. In particular, I reconstruct the fictional world (diegesis) of BrCTs – one in which CSR and social contracts have failed – before identifying eight different types of actors as which people can morally situate themselves in their relationships with business. Finally, I elaborate on the actors’ performances and their use of external and legitimate forces to end the story. The paper concludes with a discussion of potential future research to help combat BrCTs, as well as a call for the critical study of political CSR.

“I now had a greater appreciation for the celestial war between ‘good and evil’ […] I came to realize that much of our modern consumerist, money-driven society, including television, media, entertainment, industry and food production, were all the byproducts of this self-serving Orion orientation. Even if most of the CEOs of our corporations were not aware that the Orions were assisting them, they would essentially call on their services the more they indulged in their profit-motivated behaviors.”

(David Wilcock, Wanderer Awakening)

“Conspiracy theorist: a term to discredit those who have seen through the bullshit”

(David Icke, davidicke.com)

## Introduction

According to Moscovici (1987, p. 153), “[the twentieth century] established conspiracy as a system of thought and a method of action.” Aaronovitch (2010, p. 3) even states that we have entered an era of “fashionable conspiracism.” Unfortunately, how to fight conspiracy theories remains a difficult question as research outside business ethics shows how difficult it is to debunk these theories and how attempts to debunk a theory often become, in the eyes of the conspiracists, evidence that the conspiracy is real (Sunstein and Vermeule 2009). In the field of business, the question might be equally difficult to answer as companies are places where profit-motivated interests can easily win over ethical decisions, and both companies and governments sometimes participate in a cult of secrecy that contributes to the nurturing of conspiracy thinking (De Maria 2006).

It is important to study conspiracy theories (CTs) and, more specifically, business-related conspiracy theories (BrCTs) in the field of business ethics for at least two reasons. First, they threaten most of the business stakeholders: consumers and society at large, companies, as well as non-governmental and (inter)governmental institutions. For example, according to the World Health Organization (WHO),^1^ the lack of vaccination as a result of conspiracy beliefs was one of the Top 10 threats to global health in 2019. But vaccination is only one issue among many others, as research in health psychology has shown that conspiracy theories may have detrimental effects on health decisions, political engagement, and pro-environmental behavior (Jolley and Douglas 2014a, b; Thorburn and Bogart 2003). Additionally, organizations should consider BrCTs that attack them as a serious threat since they can potentially ruin the benefits of their ethical discourse and actions, like they can be warning signals of larger and less marginal negative sentiments transforming into hardly controllable consumer negative actions such as boycott, donation restrictions during fundraising campaigns, or negative word-of-mouth that spreads fake news. Finally, BrCTs question the very existence of corporate social responsibility (CSR), in particular by portraying economic agents as being unable to meet society’s fundamental demand: to be profitable while complying with the law (Carroll 1991).

Second, through their narrative form, BrCTs provide access to what Jacobs (1991) and Seabright and Schminke (2002) refer to as the moral and immoral imagination of a segment of the population. This moral and immoral imagination, which is generally difficult for researchers to capture, suggests how part of the population perceives the business world or how the latter integrates into that world based on moral judgments. Stories almost always incorporate ethical concepts, and an understanding of these concepts often relies on metaphors embedded in these narratives (Johnson 1994). As Michaelson (2005, p. 359) argues, “it has become quite common to use stories to make moral sense of business life.” Therefore, I argue in this paper that belief in a BrCT is not necessarily the simplistic result of pathological minds engaging with irrational and paranoid ideas (Hofstadter 2008). This assertion is essential because using pathology to explain BrCTs may lead the population and decision-makers to think that it is normal (i.e., the norm) to consider conspiracy theories as totally delusional and that they should, therefore, ignore them. Behind an eccentricity that some would disdainfully ignore, BrCTs are all built on an ethical reading of the world and suggest some common ethical positions that should be seriously considered.

In the absence of dedicated business research on the subject and given what I have written above, this paper has two objectives: (1) to understand, from a structural, narrative point of view, what leads people to latch onto BrCTs and (2) to decipher the ethical reading proposed in BrCTs. For the sake of clarity, I consider narratives as representations of a series of events manipulated through discourses (Onega and Landa 2014) and define a BrCT as.

an alternative, explanatory, non-refutable, and logical narrative that relates to an event or a series of events connected to a brand, company, or industry and is rooted in the belief that nothing happens by accident and that there must be a secret and/or powerful group of people pulling the strings behind the scenes to take money, freedom, power, or knowledge away from another group of people.

Like any narrative, a BrCT relies on the staging of a scene and a series of actions. By reconstructing the scene and identifying the characters and their actions, it is possible to reach a better understanding of why and how BrCTs appeal to many people, as well as the ethical positions, contestations, and deliberations that transpire from these narratives.

Drawing on Aristotelian virtue ethics and using structural narratology, I show that BrCTs are, above all, micro-narratives in which people and organizations can play roles that vary based on their ethical positions. These roles are inspired by latent macro- and meta-narratives that recount a similar story taking place in the neoliberal economic system and involve a lack of virtue, illegitimate domination, and inequality in society. The contributions of this paper are threefold. First, it introduces the concept of BrCT in business ethics and identifies a frame of reference common to the BrCTs studied. Doing so could trigger future research on the topic and hopefully help to combat BrCTs. Second, the paper sheds light on BrCTs’ particular perspectives on interpersonal relationships, social contracts, and human nature, which helps to understand the current critical debate surrounding the limitations of CSR. Finally, this paper contributes to the discussion surrounding the value of narratives and storytelling when analyzing CSR as a contested and socially constructed concept. While our understanding, construction, and interpretation of ethics are based on narratives (MacIntyre 1981), and business ethics is the result of narrative practices (Phillips 1991), business ethics journals have published very few narratological studies. Moreover, as Campbell and Cowton (2015) show, it is surprising that quantitative approaches have come to dominate research in business ethics because the issues at the heart of the field are essentially qualitative (e.g., questions of right, justice, fairness, decency, or equality).

The remainder of the paper is organized as follows. The section below theorizes belief in a BrCT from an ethical perspective, notably by highlighting the concepts of virtue ethics, ethical distress, and ethical blindness in light of the current business environment. I conclude the section by suggesting that a good fit between the frame of reference common to BrCTs, on the one hand, and ethical distress and blindness, on the other hand, can be a reason why people move from a perceived lack of virtue in business to a belief in BrCTs. To highlight this common frame of reference, I introduce the narratological study that was conducted with multimedia data retrieved online. This is followed by a presentation of the content and structure common to BrCTs – specifically, the fictional world depicted in a BrCT (i.e., the diegesis), the actors populating this fictional world, and their performances. I end the paper with a discussion that offers a few directions for future research on BrCTs and political CSR.

## Theorizing Belief in BrCTs from an Ethical Perspective

**Fig. 1:**
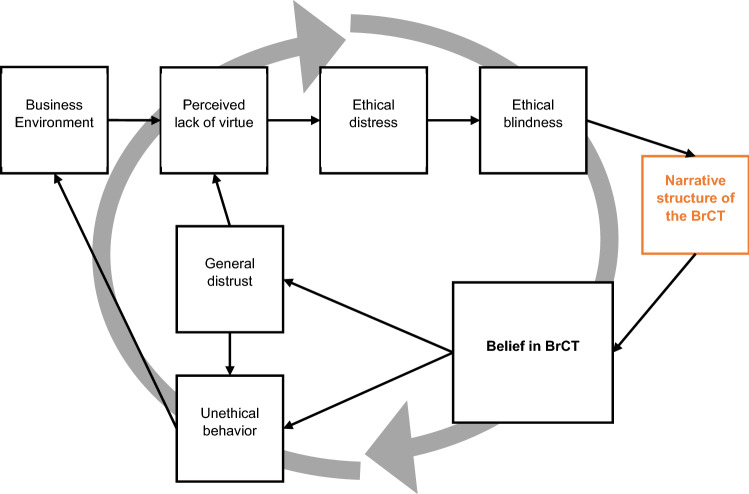
Theorizing belief in BrCT from an ethical perspective

Drawing on virtue ethics (Aristotle 2009), I theorize a BrCT belief as the possible outcome of ethical distress and blindness due to a perceived lack of virtue in today’s business environment (see Fig. 1 below).

### Virtue Ethics and BrCT

Compared with utilitarians and deontologists, virtue ethicists emphasize (among others) the critical role of individual character traits in choosing ethical actions. This means that doing good can depend on universal rules and codes that are imposed on us but also, in large part, on our individual and natural tendencies, which can be positive or negative and are influenced by the environment (MacIntyre 1981). While some Aristotelian ethicists see business as merely satisfying a desire for maximizing profit, which can prevent agents from living the good life (MacIntyre 1981), others believe that companies can be viewed as creators of opportunity to form communities with a moral responsibility and a commitment to doing good (Hartman 2011; Moore 1999; Solomon 2003). In the latter scenario, a business is considered a community of practice^2^ whose virtuosity flows in part from managers who are devoted to excellence and focus on “those internal goods thereby obtainable, while warding off threats from […][the] inordinate pursuit of external goods and from the corrupting power of those other institutions with which it engages” (Moore 2005, p. 661). Noting the almost impossible task of developing purely Aristotelian business communities built on honesty, dependability, or equality, Sinnicks (2019a) suggests that business communities should be developed while maintaining a suspicion of inequality within the community, having minimal displays of unequal power, and putting an emphasis on face-to-face interactions and relatively equal pay.

Belief in a BrCT challenges many of the assumptions about the virtuous tendency of business agents and communities. By definition, BrCTs recount the story of egoistic agents who conspire at the expense of another group of people. BrCTs represent a lack (or absence) of integrity, fairness, trust, and empathy in business, that is, a lack (or absence) of a majority of core virtues required in a business context, whether at an individual or an organizational level (Chun 2005; Murphy 1999; Shanahan and Hyman 2003). In short, BrCTs depict a business world that has seriously failed to develop Aristotelian virtue in business agents and organizations. By depicting the business world as unethical because of agents’ personal interests, BrCTs make the absence of virtue ethics an ordinary and pervasive fact of capitalism and market economies. The possible consequences of such a view are a generalized distrust of business agents and organizations, a related lack of belief in CSR and other ethical engagements, and, eventually, the end of deontological and utilitarian act theories. People who believe in BrCTs are likely to react by reinforcing their feelings of distrust and considering CSR and other ethical engagement as a veil to hide the truth about what business really is.

Aristotle (2009) argues that practical wisdom requires virtue. Practical wisdom is a master virtue and is people’s capacity to act on knowledge of what is good or bad (Aristotle 2009). Apart from a general conception of what is good or bad, practical wisdom involves the ability to deliberate well and to act on that deliberation. In light of Aristotle’s practical wisdom, the perceived lack of virtue in business agents and organizations, as well as the belief in BrCTs, would create the appropriate context for the development of negative character traits in agents who might eventually end up with unwise practices. Regarding BrCTs, unwise practices would mean an inability to make informed and rational judgments and would lead to stronger conspiracy ideation and/or unethical practices. The relationship between unwise practices, considered to be altered thinking dispositions, and conspiracy ideation has already been investigated and empirically tested in cognitive psychology. For instance, Swami et al. (2014) show that analytical thinking reduces belief in conspiracy theories. Regarding the vaccine example provided in the introduction, conspiracists who refuse to have their children vaccinated or firmly deny the reality of climate change are likely to display unwise practices in part because of a lack of trust resulting from the perceived lack of virtues in business agents and organizations. Similarly, unethical behavior from agents within organizations would also result from perceptions of an unethical business environment that encourages people to act accordingly.

### Business Environment: Suspicious CSR and Postmodern Consumer Culture

Context is crucial for the development of positive/negative character traits and virtues/vices (Aristotle 2009; MacIntyre 1981). In the eighteenth century, Adam Smith (2003) was already warning against nefarious but necessary mercantilism and the fact that merchants are likely to come together to conspire against the public. Secrecy is also a tradition of capitalism, with some companies having great power and nurturing this culture of secrecy (De Maria 2006). Interestingly, one just has to look at some of the articles published lately in the *Journal of Business Ethics* to understand today’s business environment: Corporations can cause harm; therefore, trust is ever more critical in this age of surveillance capitalism when ethical conduct requires a struggle (Alcadipani and de Oliveira Medeiros 2019; Andrew and Baker 2019; Kaptein 2017; Levine 2019). Like the tobacco and pharmaceutical industries that illustrate the late 20th- and early 21st-century lobbying traditions, some industries have often been accused and sometimes found guilty of conspiring against the law (Brandt 2009; HAI and CEO 2012). Similarly, some industries or companies have subsidized medical organizations and research centers that seemingly prove the regular and high consumption of salt, sugar, or red meat, for example, is not harmful to consumer health (Jacobson 2005; Kearns, Schmidt, and Glantz 2016).

In such a context, Fleming and Jones (2013) argue that CSR is partly an instance of propaganda that misses the forest for the trees. While companies display their ethical labels and CSR projects, consumers learn about the emissions scandal in the automotive industry (e.g., the Volkswagen Group), horse meat that is intentionally and secretly added to frozen food to replace beef (e.g., the 2013 horse meat scandal), printer cartridges that run out of ink unusually quickly (Risley 2015), or carbon emissions trading that contributes to new modes of accumulation and ultimately prevents our world from achieving structural change and new capitalist dynamics that could reduce emissions (Böhm et al. 2012). All these gaps between what Tamminen (1992) calls *espoused ethics* and *ethics in use* can only increase perceptions of a lack of virtue in business, which is already high (Cole and Smith 1996). But they can also nurture conspiracy thinking about brands, marketing, and the overall business environment being visible outputs of politico-economic imperialism designed by malevolent entities.

Apart from failures in CSR projects and the economic system in general, it is *the context of the context* of business that may lead to the perceived lack of virtue. Consumer culture and marketing have commodified conspiracy thinking. One example is the main airport in Denver, which recently installed a talking gargoyle that says, “Welcome to the Illuminati Headquarters, I mean, Denver International Airport.” Another example is the renaming of a Spanish third division football club near Madrid as Club Flat Earth F.C., or the 2015 New York Art, Antique and Jewelry Show, where a collection of items relating to the JFK assassination, including one of the seven copies of the Zapruder film, was priced at $168,500. More broadly, people indulge in “a self-conscious and self-reflexive entertainment culture of conspiracy” by consuming movies, series, video games, and books such as The Matrix, The X-Files, 24, Assassin’s Creed, Conspiracy Theory, and The Da Vinci Code (Knight 2002, p. 6). These cultural works, mediated by consumer society, infuse popular culture with the notions of secrecy and doubt, as if to suggest that social reality might well be an illusion that allows secret decisions made by a few powerful people to remain concealed (Bell and Bennion-Nixon 2000; Kellner 2002). In addition, the success of critical theory in the twentieth century would have helped spread and legitimize conspiracy thinking in our Western cultures (Latour 2004). Authors like Baudrillard (1981), Deleuze (1990), and Foucault (1975) formulated their suspicions and opinions about hegemonic entities manipulating and enslaving the masses, regardless of whether these entities are countries (mainly the US), banks, companies, or more intangible forms like consumer society. In their own ways, all these authors perpetuated what Ricoeur (1965) calls a hermeneutics of suspicion, which we already find in Schopenhauer (1818) and later in Nietzsche (1878). Today, this logic of suspicion has become intertwined with the vox populi and is particularly visible in emerging terms (e.g., alternative facts, fake news, and post-truth) that indicate concerns about what the truth is (Baird and Calvard 2019).

### Ethical Distress and Blindness

Originally coined in the fields of nursing ethics and health care, ethical distress is defined as uncomfortable feelings and a psychological imbalance of being in a situation in which one knows what is the right thing to do but unlikely to happen for various reasons (Jameton 1984; Corley 2002). From an Aristotelian perspective, ethical distress can be understood as a hindrance to living according to our virtues. For instance, Aristotle (2009) worries about the possibility that *akrasia* (i.e., weakness of will) takes people away from virtue since they would act in a way that is contrary to their choice. In business ethics, ethical distress has been observed in employee/manager relationships (Prottas 2013) or in the case of a dissonance between an organization’s operational values and what this same organization says it endorses (Mills and Werhane 2015). In the context of BrCTs, ethical distress would appear after that individuals perceive a lack of virtue in business with no official voice publicly banning unethical practices resulting from this lack of virtue, or observe that nothing changes despite official discourses. Another possible contributor to ethical distress is CSR itself, for it shows the way to make business more virtuous while simultaneously reminding us that this path paved with good intentions is not the one systematically taken by agents—not because of a lack of means but because of a lack or a weakness of will. This feeling that business does not act according to our values and principles and that nothing is going to change for the better could trigger ethical distress and lead some to believe that this absence of change is the result of a conspiracy and powerful people that they should blame or fight. In this context, believing in BrCTs would be a coping strategy to release the tensions created by ethical distress in a similar way to how nurses use various strategies to cope with ethical distress (Corley 2002).

A possible consequence of ethical distress, and possibly a mediator variable between ethical distress and BrCT belief, is ethical blindness. It has been well studied that aversive states like anxiety (i.e., a state closely associated with ethical distress) result in maladaptive behavior due to altered cognitive capacity and a focus on negative or possibly negative events (Kessler et al. 2009). Palazzo et al. (2012) propose that everyone can experience ethical blindness, that is, a temporary and unconscious deviation from our values and principles, whatever they are. According to them, ethical blindness is more likely to arise in a situation of rigid framing (i.e., rigid mental structures used to simplify and understand reality). While these authors suggest that moral imagination should be developed to promote more flexible framing, it should be noted that a BrCT is the product of moral and immoral imagination—which seems to run counter to Palazzo et al. (2012) original idea. To overcome this issue, I draw on the Aristotelian notion of virtue as an equilibrium and propose that ethical blindness is not only a temporary and unconscious deviation from our own values and principles, but also too great a (temporary and unconscious) focus on our own values and principles (i.e., rigid framing) that eventually makes us deviate from virtue ethics. This can be the case, for instance, when we lack practical wisdom and think we are doing the right thing but, actually, are not doing it the right way. In the context of this research, a BrCT is therefore understood as partly the result of an imagination that always finds inspiration in the same frame of reference. Like Palazzo et al. (2012), I call this phenomenon ethical blindness, in the sense that conspiracists would focus only on the frame offered by the BrCT, since this frame confirms their perception of a failure of virtue ethics in business and helps them structure their thoughts as it provides meaning to a complex reality (Dean 1998; Fenster 1999; Melley 2000). This proposition echoes the work of Swami et al. (2014), who show that conspiracy ideation is not only explained by low analytic thinking but also by low open-mindedness. The occurrence of ethical blindness in the context of BrCTs also finds support in literature, which emphasizes that conspiracy thinking systematically rejects established sources of knowledge (Hardwig 1991) and necessarily relies on the magical “notion that everything is or can be connected” (Dean 2002, p. 97). In the same vein, once people start believing a conspiracy, it becomes almost impossible to persuade them that the conspiracy is unfounded and should not be believed (Nyhan et al. 2013; Sunstein and Vermeule 2009).

To summarize this theoretical frame, BrCTs can be understood as a result of both actual unethical behavior in business and a perceived lack of virtue. The consequences of their belief are a general distrust of business and specific corporations, which potentially leads to unethical behavior. Regarding the reasons why people would move from a perceived lack of virtue in business to belief in BrCTs, I have suggested that the perceived lack of virtue may trigger ethical distress and blindness: Ethical distress would lead to an unwise practice of knowledge (i.e., altered cognitive capacity), which would lead to ethical blindness (i.e., sticking to rigid framing).

In such a theorization, the role of narratives is critical as they offer a frame to hold onto, through which to morally make sense of the world, and according to which to act. Therefore, there is a need to delineate the frame that BrCTs offer and is preferred to other frames proposed by official agents. To this end, the next section presents a narratological analysis of online BrCTs to delineate the frame of reference that structures BrCTs and see which related actions are proposed to believers. For the sake of clarity, I define *online* BrCTs as business-related conspiracy narratives found online but not necessarily created online and whose events are often fragmented across different sources of information.

## Narratological Study of Online BrCTs

### Narratology, Narrative Ethics, and BrCTs

Simply put, narratology is “la science du récit,” that is, the science of narratives (Todorov 1969, p. 10). Narratology “examines what all narratives have in common—narratively speaking—and what enables them to be narratively different” (Prince 1982, p. 181–182). It studies the story, which is understood as a sequence of actions or events, and/or the discourse, which corresponds to the way the story is presented (Culler 2001; Genette 1980; Greimas 1997). In this sense, narratology is not a methodology, but a discipline with structuralism and semiotics as two interrelated approaches that are particularly well suited to narratology. The interest of studying narratives lies in their power to reveal how people make sense of their self, their environment, and the relation between the two (MacIntyre 1981). Narratology studies not only narratives but also how people are influenced by them through perception and action. In the context of business, marketplaces and organizations represent arenas of deliberation—sites of symbolic creation and contestation that rely on narratives drawn from cultural traditions (Boje 1991; Ger and Belk 1996; Goulding et al. 2009). By studying narratives found in these arenas, it is possible to understand how agents make sense of their environment and how/where they situate themselves in this environment.

Within narratology, narrative ethics is interested in the intersections between stories and storytelling, on the one hand, and ethical values, on the other hand (Adams 2008). In his attempt to understand what influences the development of virtues and vices, Aristotle (2009) considers that narratives have such a capacity because narratives always include ethical matters, whether deliberately or not (Phelan 2014). Therefore, this paper borrows from narratology and especially from narrative ethics, as I suggest that BrCTs propose narratives of a failure of virtue ethics in the business world. In other words, BrCTs are narrative manifestations of the ethical contestation and deliberation that take place within marketplaces and organizations, and whose characters and storylines can be studied with the help of narratology.

### Data Collection

The data was collected online between September 2016 and January 2018 (i.e., three full weeks, followed by one full day every 1.5 weeks on average). The main reason for studying online BrCTs is that the Internet has produced a highly decentralized form of mass communication that perverts the public discourse and helps BrCTs proliferate. In Sunstein’s (2009, pp. 82–83) words, “the Internet produces a process of spontaneous creation of groups of like-minded types, fueling group polarization. People who would otherwise be loners, or isolated in their objections and concerns, congregate into social networks.” The result is the multiplication of knowledge claims that run counter to mainstream beliefs and that claimants consider as being verified, that is, on the same level of truth as other knowledge provided by officials or science, for example (Barkun 2003).

In total, 28 conspiracy theories co-produced by the media outlets and the readers who discuss the content were analyzed. These conspiracy theories, presented in Table 1, can be seen as what van Dijk (1980) calls semantic *macrostructures*, that is, the coherent overall meaning generated by the aggregation and organization of different chunks of meaning. In the context of this research, these chunks of meaning are the different data gathered from different online sources: English- or French-language conspiracy websites, blogs, forums, videos,^3^ seven debunking websites, national and regional newspapers and magazines, and brand/corporate websites that have pages dedicated to the fake information or conspiracy cases in which they are involved (e.g., Coca-Cola, McDonald’s, Whole Foods Market, Volkswagen, etc.). In terms of traffic, the conspiracy websites visited for this research receive on average^4^ between 14,000 and more than 14 million monthly visits. Since its inception, the conspiracy website and forum *Abovetopsecret* says it has generated more than 19 million posts and more than 1 million topics, with 75% of these topics getting replies. While all these figures are already enough to understand to degree to which people have easy and direct access to potentially harmful theories, it is important to note that all these theories are also shared on the most visited websites in the world: through videos on YouTube (second-most visited website in the world) and through posts on Facebook, Twitter, and Reddit (respectively #3, #6, and #13) or Amazon through books (#4).

**Table 1:** Conspiracy narratives that were studied in this research

Perpetrator(s) of the conspiracy	Direct victim(s) of the conspiracy	Narrative studied
China	Nigerian consumers African consumers	Rice imported from China is poisoned. “Plastic" rice is just one of many Chinese products imported into Africa to depopulate the continent while stealing its resources (there was, for example, a previous case of human meat sales)
Coca-Cola	Consumers	Coca-Cola plays a major role in the plans of the NWO by creating an addiction to a poison that ensures high profits at the expense of the poor population, which is kept under control
Disney	Children Consumers	The Disney brand is a tool used by the Illuminati to recruit young children and make them good citizens who will serve the elite all their lives
Enedis	Consumers Companies	The new “Linky” smart meters imposed in French homes and businesses are spies that follow all our private activities. These meters also pose health problems
Facebook The US government	Consumers	Facebook is the continuation of the DARPA project
Nestlé Water	Consumers Non-GMO farms	Nestlé is part of the NWO agenda. It is currently taking control of water, which should be free of charge. One of the reasons is that Nestlé only wants to provide water to GMO farms
Russia	Mr. Walt Disney	In the 1950s, Walt Disney considered his failures to be due to a communist conspiracy
YouTube (Google)	Consumers	Now that YouTube belongs to Google, it is at the service of Big Tech: censorship (including conspiracy theories) and brainwashing
GMO-supporting mega-corporations (e.g., Monsanto)	Applegate Farms Consumers Earth	Big Business prevents companies like Applegate from doing good and serving the well-being of people, animals, and the planet Consumers should buy local products in local stores that are themselves the victims of mega-corporations
Campbell	Consumers	Campbell cannot be honest when it says it will be GMO-free. All Campbell wants to do is make money, as illustrated by the significant donations it has made to oppose the labeling of genetically modified food products
Chocolate brands	Consumers	Most chocolate brands have dangerous levels of heavy metals that are deliberately hidden
Elves, USA, UK, France, North Korea, Uyghurs	Malaysia Airlines Malaysia Airlines passengers	What happened to flight MH370 was not a simple accident but the result of a conspiracy. It is impossible not to find an aircraft that is about 60 m by 60 m with all the technology we have today
GPS brands NASA	Consumers	The Earth is flat, but NASA wants people (including airplane pilots) to think it is a globe. GPS brands are playing NASA's game to keep selling devices and maps
Greenpeace	Resolute Resolute’s customers	Greenpeace deliberately falsified and published photos and videos to harm the Canadian company Resolute. These photos were intended to show how much Resolute harms forests, while these photos show forests that have been devastated by fire or other natural causes
Kleenex	Consumers	Kleenex tissues have a powdery substance that they use to cause dust in your nose and make you use more tissues
NGOs The beef industry	Consumers Earth	NGOs make money from the beef industry instead of fighting for the environment
Whole Foods Market	Consumers	Whole Foods Market deliberately misleads its customers through its communication and training of its employees who learn to lie and say that their stores sell "Nothing artificial, ever," when in reality they sell products with GMOs and conspire with Monsanto
Apple	Consumers Companies	Apple removed the headphone jack on the iPhone 7 to better control consumers through a DRM audio scheme. Apple responded to this charge by saying that “It has nothing to do with content management or DRM—that’s pure, paranoid conspiracy theory”
Big Tech	Consumers Governments	Big Tech is now Big Brother. It decides everything and can censor all truth, use people's telephone microphones, monitor and follow people and governments. Scientific and technological elites such as Google are pursuing a transhumanist agenda with the help of consumers, which will lead to a technologically advanced ruling caste benefiting the NWO
Companies Green activists	BP and the oil industry Consumers	Global warming is an invention. The oil-rig industrial accident in 2010 in the Gulf of Mexico, like other accidents, was caused by eco-warriors and supported by all corporations with an interest in promoting environmentalism
Google (Alphabet)	Consumers	Google is one of the leading agents of global authoritarianism through the control of information (and, thus, the mind). Its different logos reveal that it is a devil worshipper
Indian government	Maggi (Nestlé India) Indian consumers	The Food Safety and Standards Authority of India (FSSAI) has asked Nestlé to recall its Maggi 2-min instant noodles. The real reason is that Maggi is loved by the Indians – as shown by its 75% market share, which prevented Baba Ramdev from successfully launching his instant noodles (Patanjali). Therefore, the Indian government decided to intervene in favor of a national brand
Kellogg’s	Consumers	Kellogg's wrongly says that it does not use GMOs, antibiotics, or pesticides in its recipes to please consumers and make profits that are donated to George Soros's Open Society Institute
Pokémon Consumers	Consumers	Pokémon products are covered with Illuminati signs and are designed to control people by teaching Illuminati’s agenda from childhood. As a result, an “army” of consumers is well trained to teach how to serve the elites
Snapchat	Consumers	Snapchat does not receive billions of dollars from investors just because it is a good application. Behind the scenes, it takes 3D scans of all human faces and stores them to better watch the population
Big Pharma	Consumers	Pharmaceutical companies are pressuring the Food and Drug Administration (FDA) to annihilate natural remedies for cancer. In addition, major pharmaceutical companies have been involved in the creation of conditions and diseases such as AIDS, Ebola, and ADHD. Vaccines are a farce and should be avoided
Western brands Non-Western consumers	Non-Western Consumers Non-Western countries and cultures	Western brands, especially US ones, are exported to dominate the world. For example, Western food companies use GMOs to alter the DNA structure of Muslim consumers, Pepsi and Coca-Cola steal water resources from the local population, and films and comics infuse materialistic and imperialist ideologies through a priori harmless entertainment The problem is that consumers themselves, by promoting Western products (e.g., by selling and buying American films on the Iranian black market), are acting against their own interests. Western brands and products should be boycotted by all
Monsanto/Bayer	Consumers Companies Governments	Monsanto has long misled everyone, but today's protests and guilty verdicts at jury trials highlight its true nature

When dealing with online data collection, Markham (2003, pp. 53–54) reminds us that one “must read the text and interactions of interests, much like trail signs, and make defensible decisions about which paths to follow […] and therefore which boundaries to draw.” She adds that these boundaries would be better if derived from the context than a priori guides and criteria (Markham 2003). In line with her guidelines, the data was collected according to three criteria chosen after the first round of observation: the type of perpetrator (e.g., company, brand, industry, government, association, consumers, etc.), the type of victim (e.g., children, consumers, companies, company, brand, industry, government, the planet, etc.), and the integrative theme of these narratives (i.e., control, rebellion, deception, or a combination of them). These three criteria were the result of preliminary analysis objectives that consisted of determining the semantic macrostructures, as well as the key elements that structure them. As we can see, these three criteria are close to some of the most critical “ingredients” for a narrative to exist in narratology: characters and their functions, as well as the object guiding their actions (Greimas 1983).

One of the problems encountered during data collection is that the information posted on the websites is not always false or necessarily related to a CT. In fact, these websites often mix reliable and unreliable information. Therefore, to decide whether the narrative was a CT, I considered (1) the source (i.e., website listed as conspiracist or not, according to a list provided by debunking blogs or the national French media), (2) whether it was explicitly mentioned that it was a CT—for instance, when narratives were found in local or national newspapers, (3) whether the narrative fit the proposed definition of a BrCT, and finally, (4) whether conspiratorial rhetoric was used. Rhetorical elements include evidence (i.e., proof is all around and should not be questioned), emotion (i.e., one should be afraid of conspirators and be aware of how damaging conspiracies are to humanity, the planet, etc.), belonging and exclusion (i.e., it is me and us versus them), critical thinking (i.e., reasoning, searching for proof, questioning official discourses and apparent truths…). The number of articles, videos, and posts used for this research was based on the principle of saturation, which means that content for each conspiracy narrative was scrolled through as long as the structure and content of the narrative remained unclear to me and finished when I reached redundancy.

### Structural and Semiotic Analysis of Data

Structural narratology is particularly well adapted in the case of BrCTs because conspiratorial rhetoric has kept its style over time—some even suggest there is a *tradition of explanation* (Billig 1978). For example, the conspiracy narratives about the 2008 financial crisis are the same as those about the Great Depression (Byford 2011), and the 9/11 Truth movement uses interpretive frameworks similar to those drawn by conspiracy theorists who accused President Franklin D. Roosevelt of letting the attack on Pearl Harbor take place in order to force the USA’s hand and have the country enter World War II (Kay 2011). In other words, despite the variety of events and the significant changes the world has undergone over the years in terms of communication tools and channels, the structures of CT have remained constant.

Structural narratology relies on several assumptions (De Saussure 1916/2011; 1976). First, it is possible to discover the underlying principles that compose, structure, and provide meaning to a narrative or group of narratives. Second, texts have abstract levels that should be taken into account. For instance, Barthes (1957) shows how media convey meanings within popular culture that are partly based on ideologies and deeper than the formal meaning that is immediately given to us. Third, a narrative can be understood through two pairs of axes: paradigmatic (e.g., the event in itself) and syntagmatic ones (e.g., the sequence of the events). As these assumptions highlight, structuralism is intimately related to semiotics (Lorusso 2015), that is, the study of “the life of signs within society” (De Saussure 1916/2011, p. 16). For De Saussure (1916/2011), one of the early fathers of structuralism, the sign is at the heart of the language system. Going back to narratives, this means one cannot fully identify and analyze a narrative’s underlying principles if one is unable to decode what the signifiers stand for. In this regard, one major structuralist tenet is that characters and storylines structure texts and semiotic relationships. Characters are textual beings—unifying tools that gather a mass of signs and symbols (Mick 1986).

Data analysis was carried out following the general rules of structural semiotics. The aim was to analyze how BrCTs can be understood as the result of implicit and, therefore, difficult-to-grasp overarching narrative structures that shape (and are shaped by) ethical behaviors and beliefs, as these same structures provide indications of people’s perceptions regarding ethics in the business world. First, the different versions and contexts of the same BrCT were compared with each other, based on Levi Strauss’s (1976) methodology. Second, a referent version of each BrCT was identified based on its occurrence, and a set of notes previously taken for each narrative available was used to outline macrostructures (van Dijk 1980). For instance, BrCTs about Chinese products imported to Africa do not necessarily propose the same protagonists, actions, or objects of action. In the end, the macrostructure of the referent version that was retained is that *Chinese rice imported to Africa is poisoned on purpose to depopulate Africa and steal its resources*. Third, the 28 BrCTs were coded in the tradition of structuralist narratology, that is, to identify codes or sign systems that provide frames of reference (Jakobson and Halle 1956). As Spiggle (1998, p. 167) reminds us, “the frame arises from inferential efforts as the researcher moves back and forth between analyzing data constructing interpretation.” Based on the readings made in the previous steps, which mainly highlighted a binary worldview that structures the narrative elements (e.g., good or bad, victim or guilty…), coding was done by using Greimassian (Greimas 1982) semiotics and 1976 structural study of myths, which takes account of both the paradigmatic and the syntagmatic axes of sign systems. As an example, based on the Greimassian actantial model, a series of codes regarding personal ethics, worldview, actions, and social position were created for the different protagonists. Similarly, codes about location, time, or consequences were created for the events. Axial coding (i.e., relating categories) was done through relations of opposition (e.g., categories *A* and *B*), complementarity (e.g., categories *A* and *not B*), and contradiction (e.g., categories *A* and *not A*), as found in the work of Greimas (1983), among others. In parallel and based on Lévi-Strauss’s work (1976), actions in the narratives were structured along syntagmatic and paradigmatic axes. This means that attention was given not only to what composes these actions and what they mean (i.e., paradigmatic axis) but also to the order in which they happen (i.e., syntagmatic axis). In the Chinese rice example (and, more specifically, the Nigerian case),^5^ this means that “Nigerian Customs announcement of fake Chinese rice,” “Nigerian ban on rice importation,” and “Nigerian complaints” should be differentiated because of their content (e.g., information per se, protagonists…) and meaning, but they should also be evaluated with regard to the sequence of these events (e.g., the meaning of the narrative is different depending on whether, for instance, “Nigerian ban on rice importation” happens before or after “Nigerian Customs announcement of fake Chinese rice”).

Finally, the BrCTs were re-read in light of the elementary structures of meaning found in the previous step so as to better delineate Souriau’s (1951) *diegesis* of these 28 BrCTs. While the term *diegesis* was already used in Ancient Greece, Souriau (1951) gave it new life in film studies before it became well used in narratology, notably by Genette (1997). Diegesis is understood as the visible and invisible universe triggered by the narrative and supported by the movie. In other words, it is everything that could happen or exist in this fictional world that the spectator or filmmaker can imagine (Souriau 1951). In the context of this research, the diegesis is defined as the world that is explicitly and often implicitly (i.e., through *indices*, see Barthes 1986/1994) referenced by BrCTs; it is *what* sets the stage. The aim of this final step was, therefore, to better comprehend which worldview(s) conspiracists adhere to when they adhere to business-related conspiracy narratives that potentially shape the conspiracy narratives structures that were found.

## Findings: Deceitful CSR and Broken Social Contracts

### Mise-en-Scène: The World as Depicted by BrCTs

In theater and filmmaking, the *mise-en-scène* is the setting of the stage in such a way that spectators can better identify the world the characters inhabit. The mise-en-scène enables the creation and presentation of the diegesis, that is, the fictional world that spectators can imagine. In literature, this mise-en-scène is mainly carried out through description, which has the same role in creating the diegesis and helps readers explain and justify the psychology of the characters, as well as their actions (Genette 1997). In this first section of findings, I draw on the data collected to develop this fictional world, which helps readers ethically make sense of the different events and characters recounted in a BrCT. To facilitate comprehension, many of the verbatims and examples I provide come from the same BrCT associated with Enedis’s smart meters.

*Human Nature is Evil: Myths of Domination and Instrumental Capitalism*. BrCTs can be read at a micro-, macro-, or meta-level that jointly depict and explain CSR issues. As we will see in the paragraphs below, micro-narratives often deal with particular CSR failures. Macro-narratives explain these failures by pointing to systemic issues, while meta-narratives support the explanation by providing arguments related to human nature. For instance, consider the following verbatim about the French electricity company Enedis, which was posted in 2017 on a blog for people opposed to the smart meter:

Courts and lawmakers are no longer independent, the lobbies will always win.

**Fig. 2:**
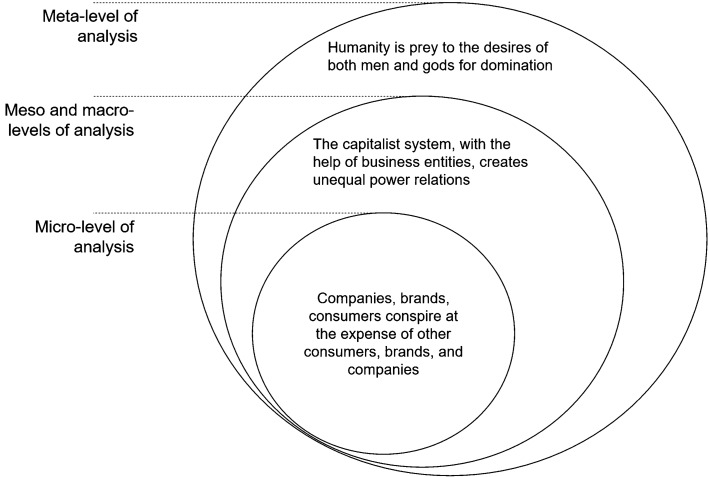
Multi-level reading of BrCTs

This sentence can have at least three interrelated meanings corresponding to the three levels of reading presented in Fig. 2. At a micro-level, it simply highlights the fact (according to the author of this post) that justice and business work hand in hand at the expense of consumers. At a macro-level, it stipulates that justice is supposed to be independent in our societies, remain partial, and protect the population. But today, for financial reasons, justice has been corrupted and our societies suffer from unwanted *taboo trade-offs* (Fiske and Tetlock 1997)—we will discuss these taboo trade-offs in the next part of this section. At the meta-level of reading, the sentence corresponds to and reinforces the age-old myth of domination and power, according to which the world is made up of dominant and dominated people, and those who have power “will always win.”

At a micro-level, BrCTs use very general rhetoric and reproducible content about CSR failures across five main topics: (1) lies from the organization/brand about the products’ recipe or content, (2) lies from the organization about its real intentions/strategy/values, (3) slavery and manipulation, (4) alliances (between different organizations, between organizations and governments, between organizations and consumers, etc.), and (5) the unfair “David vs. Goliath” fight, e.g., SMEs fighting against multinationals or other international organizations. This means that, regardless of what the allegedly conspiring organization is, the micro-narrative will always involve more or less general rhetoric about one of these five topics. For instance, one could slightly revise the following verbatim about the Enedis smart meter case to have a new conspiracy theory not related to Enedis at all:

They lie to us, obviously. Everything is much more complex because we are in another physical, scientific, and politico-economic dimension, which on this scale (35 million captives) is totally new.[…] Linky is the cornerstone of a new model of society that is being put in place […] We have been working on Linky for over eight years, and we know more than the Enedis staff, who know nothing except the businessman strategies that must be implemented. (source: comments in a forum from R., who presents himself as an Enedis subcontractor—2016).

By simply replacing “Enedis” with “Big Pharma,” and “Linky” with “vaccine X,” a new conspiracy narrative opens a vast world of deception and control. From this general and reproducible content, some more specific information is then added regarding the particular BrCT. This specific information enriches the narrative with more concrete events and proof that potentially make the narrative easier to latch onto, especially in the case of ethical blindness. In the Enedis case, for example, one can move from abstract theories of “another physical, scientific, and politico-economic dimension” to concrete specificities of the smart meter:

Enedis forces us to install smart meters in our homes to watch us 24/7 and make us sick at will with their radio waves […] Every 10 min, Linky can report the power consumed, which means that it can tell whether you switch on a lamp, an oven, a washing machine – in other words, whether someone is at home… (source: article comments from D.P. – 2017).

By providing concrete and more precise information, this micro-level of reading is critical because it is central to the belief of and immersion in the world a BrCT offers. For instance, the information provided about smart meters in the verbatim above offers readers the opportunity to project themselves into this narrative because they also have a home, a washing machine, a lamp, etc., and can then imagine how Enedis potentially spies on and controls them. Just like it is shown that consumers can be transported more easily into a narrative world through self-referencing and then pay less attention to the weak arguments of the narrative (Escalas 2007), this micro-level of reading may operate according to the same logic: to help readers penetrate the fictional world through self-reference and adhere more easily to the theory offered.

These micro-narratives are tainted by a macro-context of evil and neoliberal capitalism. This is the case, for example, with this comment retrieved from a conspiracy website:

the devil’s greatest trick is convincing the world that he doesn’t exist. Neoliberalism is quite similar in some respects. Under the guise of humanitarian values; racial, economic, and sexual orientation equality, social justice, etc. – the policies of the institutions that push for them quite often work to subvert the very causes they profess to want to strengthen. It amounts to nothing less than totalitarian social engineering for fun and profit. (L.B. – 2016).

While conspiracy narratives are often the result of a more latent view of what the system is, this system is capitalism with the marketplace as its best representative in the case of BrCTs. These narratives recount the story of unequal power relations between, on the one hand, Big Business, international organizations, and elites in general, and on the other hand, so-called ordinary people and small companies. By and large, business-related conspiracy narratives depict capitalism as an international corporate command-and-control system that allows wealthy people to get richer at the expense of the poor. Capitalism provides tools for domination and is the result of a simple equation: Capitalism needs many of the poor to make a few rich. The business world (notably, through brands and companies) becomes the symbol of a capitalist system that has imposed its own rules on the population to create unequal power relations, as well as physical, social, and cultural domination.

This macro-environment and the many criticisms of capitalism have to be understood through the following meta-narrative: The need for domination is inherent in humans, and capitalism provides objects of domination. Generally speaking, meta-narratives reveal central, all-encompassing ideas considered to be universal principles about interpersonal relations, human existence, metaphysics, and, more broadly, everything that can help to socially structure societies and explain the order of things. Within meta-narratives, mythological meta-narratives are contemporary discourses about humanity based on myths, past events, or they are stories that assign meaning to a possible future. While the meta-narrative can change slightly over the years, the core idea is mythological and stays the same. In our case, BrCTs underline a mythological meta-narrative about humanity falling prey to both men’s and gods’ desire to be dominant. This mythological context of domination is an enduring topic supported by various myths and ideologies that are ingrained in our cultures—to the point of developing a theory of social dominance in social psychology (Sidanius and Pratto 1999). For instance, mythological narratives teach us that the gods themselves have fought to reach their dominant positions, while humans have sought to speak out and fight back against the gods’ domination. Pagán (2004) points out how many conspiracy theories in Roman narratives included the struggle for freedom from slavery, that is, the opportunity for excluded groups to play a critical role in the world.

This timeless narrative about domination not only shapes macro-narratives regarding the way capitalism is understood but also validates the hypothesis offered by a specific BrCT: While proposing facts that supposedly happened and would prove the existence of a conspiracy, a BrCT implicitly uses mythologically founded elements to validate its hypothesis (e.g., it is in the nature of men to act this way, and since some conspiracies have already been revealed to be the truth, the conspiracy is probably real). Therefore, from an examination of the sentence, “Courts and lawmakers are no longer independent, the lobbies will always win,” we can deduce that lobbies “will always win” is understandable and believable because humans are inherently searching for the dominant position, which triggers more vice than virtue and necessarily makes “courts and lawmakers no longer independent.”

#### Internal and External Domination through Taboo Trade-offs

A diegesis also provides information about the types of relationships that actors can expect in the fictional world. In the case of BrCTs, the perception of a world dominated and controlled by a few powerful individuals and organizations is strengthened by remarks about how social contracts are mostly broken between organizations and consumers, and, to a lesser extent, between consumers themselves, or between organizations. The perception of broken social contracts in this fictional world is possibly a cause of ethical distress and a lack of faith in business ethics as it indicates the failure of contractualism and the victory of a Hobbesian state of nature where, in line with myths of domination, we do not owe each other anything—unlike Scanlon’s (1998) ethics, where it is *the war of everyone against everyone* (i.e., *bellum omnium contra omnes*—Hobbes 1651/1996). These broken social contracts, such as they appear in BrCTs, can be better understood through the lens of Fiske’s (1992) theory of social relations. According to Fiske (1992), four elementary relational models co-exist within a group of people: communal sharing, authority ranking, equality matching, and market pricing. While any of these four models can be chosen or combined for a given society, problems arise when members of society violate one or more of the values that regulate that particular society’s relational schema. These violations are what Fiske and Tetlock (1997) call “taboo trade-offs.” In the case of BrCTs, they are the product of internal and external domination.

Domination can arise from an at-risk society or from the outside. Internal domination results from taboo trade-offs by actors in the current relational schema (e.g., companies in a given society that conspire against companies or consumers of the same society). It forms part of a worldview in which the liberal logic of capitalism—and of consumer society, which promotes market pricing and authority ranking types of relational agreement—is opposed to (and often takes the upper hand in) the communal sharing and equality matching types of relational agreement collectively decided by democratic societies. For instance, the Enedis conspiracy theory proposes that people’s fundamental rights to electricity and property are no longer respected (e.g., “The right to electricity is a fundamental right”; “even respect for private property, one of the foundations of the declaration of human rights in France, is no longer respected”) because Enedis and business in general can do whatever they want, including deceive, without being punished (e.g., “What will prevent Enedis from tampering with meter readings? Their good word? Like the one given by car manufacturers on CO_2_ emissions?”; “Enedis’s decisions reveal a great deal about its desire to proceed by force and about its sense of impunity”). More generally, conspiracy narratives underline how the marketplace’s relational schemas have won out over their democratic counterparts, thereby braking social contracts between governments and their population. They also emphasize how social contracts have been broken between society and business, with marketplace actors making us believe that the market is the result of a democratic desire, a journey toward individual freedom, while the final objective is total control over the population and the establishment of a pure authority ranking model of social relations. For instance, in the eyes of the conspiracists, Whole Foods Market sells food with GMOs and secretly deals with Monsanto to support the deregulation of GMO crops, thereby deceiving people by using the language of healthy eating and sustainability to make consumers think the company is acting in the interest of society and the planet. For those who believe there is a conspiracy, the real story is that Whole Foods Market plays on the same team as Monsanto and that the system to which they belong makes consumers believe they have a choice about the way they live (e.g., deciding to consume organic food or not) when they actually do not.

External domination can result from alternative relational models coming from other societies that (sometimes abusively) compete with the existing model of the at-risk society (e.g., companies that conspire with governments and international organizations to expand their territories and impose their relational schema at the expense of alternative and local relational models). These narratives of external domination are often deeply rooted in cultural, geopolitical, or religious issues. For example, in 1994 in India, Hindu fundamentalists boycotted Coca-Cola and Pepsi out of fear that a flow of Western products would lead to new colonial domination. In Iran, some people believe that those who sell and buy American movies on the black market are part of a vast conspiracy that will benefit American sovereignty. Similarly, some believe that American comic book characters are used to inject the materialist/imperialist ideologies of the US by broadcasting seemingly harmless entertainment or that Western food companies use GMOs to change Muslim consumers’ DNA structure while Israel reaps the benefits of the money of American companies like Coca-Cola, Disney, and McDonald’s to dominate the Middle East. While many American companies are targeted, Chinese companies are also coming under fire as their business activity becomes international and more visible. For example, in Nigeria, there is a conspiracy narrative alleging that the Chinese government intends to control and sometimes even kill not only Nigerians but the entire African population through its companies’ investment activities on the continent. Whether this takes the form of poisoning the population, spreading hidden propaganda through the media, or becoming increasingly dependent on Chinese products, the motivations for such conspiratorial actions are always related to the meta-narrative of domination desires and a fear of its possible consequences.

### Eight Actants on a Manichean Stage

In this fictional world presented above and made up of those who dominate and those who are dominated, BrCTs give individuals the ethical task of situating themselves as a victim (i.e., a good person *but* a target of the conspirators) or as a guilty party (i.e., a bad person *but* a beneficiary of the conspiracy). In sum, BrCTs enable individuals to decide whether they would fight for a common or personal interest even as these conspiracy narratives sanction a society where people selfishly protect their own interests at the expense of the common good.

**Fig. 3:**
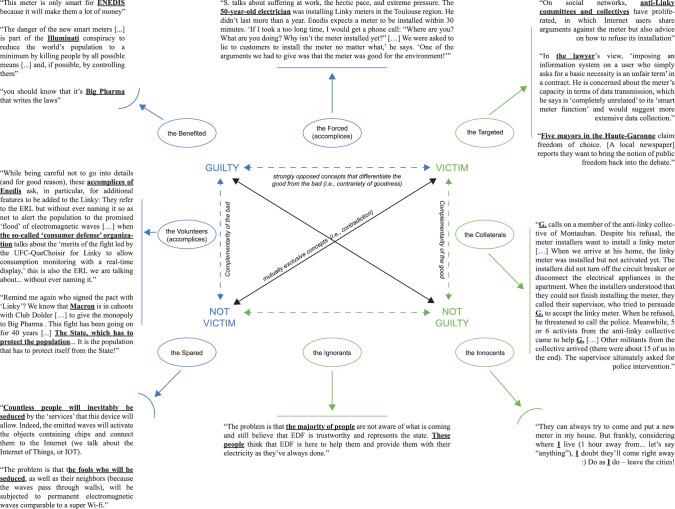
Greimassian semiotic square of the Guilty/Victim Relations—Illustrated with the Enedis smart meter case

**Fig. 4:**
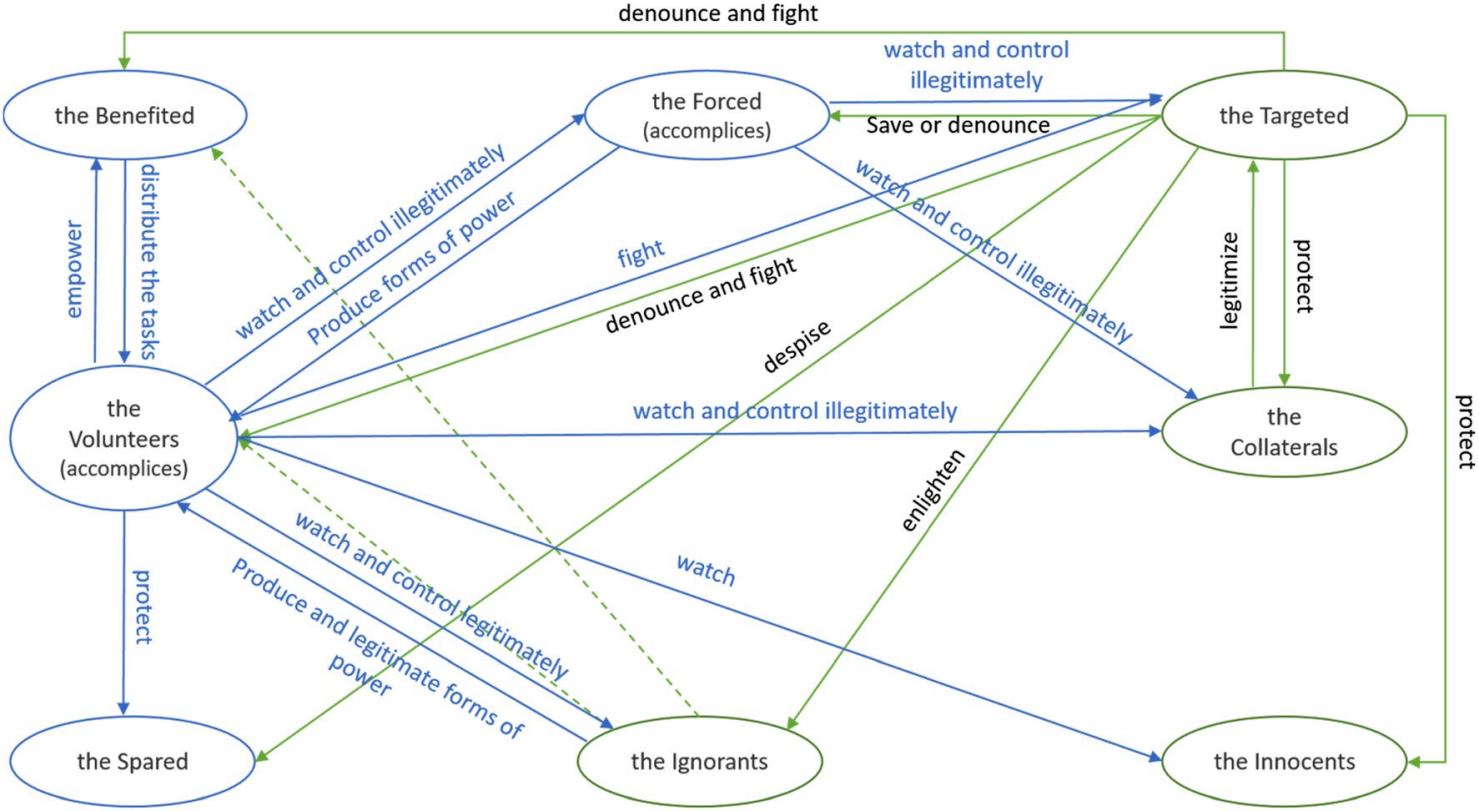
Proposed Elementary Structure of Meaning and Action in BrCTs

BrCTs propose eight different functional types of actors*,* called *actants* (Greimas 1983), and their role in the narrative. These actants help to simplify the complexity of reality, which makes them particularly attractive to people who are ethically blind due to ethical distress. As shown in Figs. 3 and 4, these actants and their roles in the narratives are structured around a Greimassian semiotic square that opposes the terms “victim” and “guilty” and differentiates the good from the bad (in green and blue, respectively, in Figs. 3 and 4). These roles, presented below, are precisely those that BrCT readers and believers can symbolically (or not) take on through self-referencing.

#### The Benefited Actant (Considered to be the Guilty Party of the Conspiracy)

The Benefited actant is located at the source of the conspiracy and perceived as the ultimate beneficiary. It often takes the form of a secret group of people pulling the strings behind the scenes. This actant’s long-term objective is to ensure the general population will be kept in check and remain Ignorants to accomplish routine tasks that generate wealth and perpetuate unbalanced but beneficial power relations. At a micro-level of analysis, an actor or character of this actant can be a particular company. For instance, according to Apple, the removal of headphone jacks is the result of improvements in the way audio is delivered, when “in fact” there are secret ulterior motives behind that decision that can be traced back to Steve Jobs himself. According to those who believe this conspiracy theory, Apple seeks to go further in controlling consumer behavior by installing a digital port that will be locked down with DRM schemes. The ultimate beneficiary would be Apple itself, which will oblige its customers to buy only expensive Apple-certified accessories and prevent people from playing a song that they should own because of content management or DRM issues. At a macro-level, the Benefited can take the form of international organizations and members of a club, such as the UN or the Bilderberg Group, or entire nations. For example, Coca-Cola—among other US companies—allegedly takes money from consumers all over the world by selling addictive soft drinks to help Israel expand its territory and succeed in establishing a global Jewish hegemony, as evidenced in The Protocols of the Elders of Zion. This narrative gives almost full credit to Israel as one of the main actors of the Benefited actant.

Although the Benefited actant is at the root of the conspiracy, the existence of helpers of the Benefited highlight that the Benefited rarely succeed alone in a narrative and have to confront enlightened people who become aware of the conspiracy and may decide to protest. As shown in Fig. 3, the Benefited will be actively helped by three other actants (i.e., the Volunteers, the Forced, and the Ignorants), passively helped by two actants (i.e., the Spared and the Innocents), and opposed by two actants (the Targeted and the Collaterals).

#### The Volunteers Actant (Considered to be Both the Guilty Party and not the Victim of the Conspiracy: Deliberate Collusion)

This actant serves the Benefited. It works for a better personal future at the expense of the collective good, even though its actors or characters sometimes convince themselves their actions are for good reasons. These actors are perceived as the elites (e.g., CEOs, heads of state, etc.) working inside the trinity of politics, religion/finance, and business.

#### The Forced Actant (Considered to be Both the Guilty Party and the Victim of the Conspiracy: Forced Collusion)

Unlike the Volunteers, the Forced actors serve the Benefited against their will. They can be companies that are required to divulge private data (e.g., Google with the NSA when it is not considered an actor of the Volunteers or the Benefited) or employees who have no other choice but to work for the Volunteers. While these actors say it is not their fault, the narratives can make them appear guilty since one is supposed to always have a choice in life (i.e., the consequence of a Manichean view on the world). Also, narratives can make this actant the object of salvation, which, therefore, should be protected and helped by the Targeted actant.

#### The Ignorants Actant (Considered not to be Either the Guilty Party or the Victim of the Conspiracy)

This actant comprises the vast majority of consumers, (often small) businesses, and workers who do not realize the harm they can do by participating in maintaining the current consumerist system. Consumer society is a tool to exercise total control over the population through brainwashing and is a central element of the Benefited’s agenda. The Ignorants actant is the upshot of this strategy of mind control and should, therefore, be enlightened by the Targeted. Ignorant actors are neither the victims nor the guilty parties of the conspiracy as long as they are not aware of the conspiracy.

#### The Spared Actant (Considered not to be the Victim of the Conspiracy)

The Spared is the group of actors who are aware of the system but prefer to accept it and play the game to live a quiet life without any trouble. The actors have the same profiles as members of the Ignorants actantial group (i.e., they actively participate in consumer society), but they know what is at stake and can identify marketing attempts to control them—this is why they are not the victims of the conspiracy and do not fall into the “not guilty” camp.

#### The Innocents Actant (Considered not to be the Guilty Party of the Conspiracy)

Similarly to the Spared, the Innocents actant is aware of the system and has decided not to fight. Unlike the Spared, however, the Innocents actors do not want to play the Benefited’s game and may end up living on the margins of society. They are diametrically opposed to the Benefited since they do not seek wealth and power and have no specific request. As long as they stay on the margins of society and do not represent a direct and immediate threat to the Benefited, they are not considered to be the victims.

#### The Targeted Actant (Considered the Victim of the Conspiracy)

This actant is the symbol of good fighting against evil. This is the group that balances the storyline. Its actors are the ones “who ask questions” (unlike the Ignorants actors, who do not contest official claims and believe easily). They “see through the bullshit” and want things to change because they can see the system’s negative impact. Therefore, they are diametrically opposed to the Spared actant, which knows but remains silent. They are perceived as the main target of the Benefited and Volunteers.

*The Collaterals actant (considered to be both the victim and not the guilty party of the conspiracy).* This actant is the group that generally collaborates with the Targeted. Its actors know about the conspiracy and want to protest but also want to avoid being identified by the Benefited.

### Actors’ Performances and Hopes for the Right Deus Ex Machina

So far, we have seen that BrCTs offer a specific, emotionally engaging view of the world through a narrative mise-en-scène, as well as eight actants that play a specific ethical role in the conspiracy narrative. These eight actants, along with the mise-en-scène, help people situate themselves in the narrative and act accordingly. In this last section of findings, I elaborate on the actors’ performances, who aim at the end of the narrative, and how many of them use a deus ex machina technique, that is, by calling upon external and legitimate forces to end the story. I use this expression since actors’ technique to end the narrative is similar to the use of a deus ex machina in the theater, which suddenly intervenes to put an end to a plot when the situation is all but impossible to resolve. But as we will see below, in the 21st-century business context, actors do not expect God to intervene; instead, they look to non-religious forces inherited from the Enlightenment that supposedly do the right thing for good: science, justice, and public institutions in general.

#### Actors’ Performances

Actors’ performances depend on people’s willingness to enter the narrative. Willing actors follow the logic of their referential actant, as shown in Fig. 4. For instance, in the case of Enedis, those who situate themselves as a Targeted actor can join anti-Linky communities and fight against Enedis by signing a petition to ban the Linky smart meter. They can also protect consumers who do not want the new smart meter (i.e., Collaterals actors) by preventing Enedis employees from entering people’s homes. Finally, they can also spread the BrCT to enlighten the Ignorants and offer them, in return, the ethical task of choosing their actant. By contrast, unwilling actors, such as Campbell or Coca-Cola, which never wanted to be perceived as a Collateral or Targeted actor, often try to end the narrative by deciding not to perform a given actant. To this end, they reproduce and strengthen the modern meta-narrative of the power of reason and logic by explicitly emphasizing the “craziness” of the situation, like Apple through Apple’s executive Phil Schiller, who declared in BuzzFeed News regarding the removal of the headphone jack that.

The idea that there’s some ulterior motive behind this move […] It has nothing to do with content management or DRM – that’s pure, paranoid conspiracy theory. (BuzzFeed News – 7 September 2016).

Another strategy for unwilling actors is to follow the actantial structure of BrCTs while changing actant. As Hamon (1972) stresses, the same actor can start the narrative by performing one actant and end up playing another actant. This is what some tech companies such as Google, Microsoft, and Apple^6^ have sometimes tried to do with more or less conviction in their relationships with the NSA and/or FBI by ensuring consumers they would protect their data as well as they can against the US government—thus moving from a Volunteer actor to a Targeted or Collateral. This shift between actants can also be made by willing actors. As shown in Fig. 3, a 50-year-old electrician can start as an Ignorant by working for Enedis for years (installing old-generation meters), become a Forced actant who does not want to install the Linky smart meters but is obliged to, and eventually decide to reveal Enedis’s “secret instructions” to everyone and act as a Targeted.

#### In Search of a Deus Ex Machina

Regardless of their performances, willing and unwilling actors often end up calling on institutions to act as a deus ex machina and end the narrative. While many conspiracy narratives create a fictional world of corrupted institutions led by the Benefited, many Targeted or Collateral actors still hope for the right institutions to do good. In the end, actors from all types of actants call upon institutions: not just Targeted actors who sue the Volunteer or Benefited actors but also Volunteer or Benefited actors who use science and national agencies to claim their innocence. The case of Enedis highlights this critical but ambiguous role of institutions, which illustrates today’s legitimacy crisis of these same institutions. On the one hand, Enedis proclaims its innocence by telling everyone it has heard about and worked on potential health and privacy issues and is supported by the government and even the European Union, which has been favorable toward these smart meters since 2009. Through its legal director, Enedis also affirms that it respects the law and acts according to a legislative framework that does not depend on Enedis. Finally, Enedis even reminds skeptical people that it is a public service company largely owned by the French state, which would prevent Enedis from conspiring against the population. On 11 September 2017, Enedis’s legal director added in the columns of the French financial newspaper *Les Echos* that the company “avoids that end as much as possible, but if it is necessary, that [they—*originally we*] have the law with [them—*originally us*].” On the other hand, anti-Linky activists also rely on the law and institutions to take on Enedis. For instance, a few mayors support anti-Linky collectives, and the latter have taken (and sometimes prevailed in) legal action. They also have a few doctors, albeit a minority—e.g., people like Dr. B., who deliver medical certificates that prevent Enedis from installing the new smart meter because of alleged electromagnetic hypersensitivity while also providing scientific reasons for refusing the smart meter and believing the conspiracy theory. By contrast, Enedis highlights reports from national agencies such as ANSES or ANFR that are based on ad-hoc studies and scientific literature. In the end, scientific and public institutions created in the era of Modernity to enlighten people about what is true, right, and good for the largest number, with a promise of protection, are today in a situation where people and organizations still hope they will find protection and ethical exemplariness in them. However, they never know whether these institutions will keep their promises in a fictional world, as we have seen above, where social contracts are broken, virtuous agents are lacking, “courts and lawmakers are no longer independent, and the lobbies will always win.”

## Discussion

BrCTs offer people a narrative world that responds well to the possible feelings (i.e., ethical distress) and thoughts (i.e., ethical blindness) generated by the perception of a lack of virtue in business. Adherence to this narrative world, perhaps even more than to a particular theory, allows one to create meaning about the world and morally situate oneself within it. Indeed, the results of this research stress that the content and structure of BrCTs take up major narrative themes that are thousands of years old yet still as attractive as ever because they not only depict the human condition but also question it. BrCTs offer a vision of the world and mankind, as well as an ethical struggle between good and evil that takes place within the market and in which every reader can take part by identifying with one of the eight actors who structure the narrative. While these themes possibly strengthen both the perception of a lack of virtue in the business environment and, by extension, ethical distress, it is important to note that it is primarily the micro-level of reading that seems to reinforce ethical blindness. Equally important, the tension created by the ethical questions posed in the BrCTs almost always awaits an end that only an external and legitimate entity can give—what I have called, by analogy, a deus ex machina. But the weakening of the state and the asymmetry of power with the business world today lead to a failure of the deus ex machina.

Considering CTs in general and BrCTs in particular as narratives opens up new perspectives on how and why they are created, spread, and believed. I am delighted to see that colleagues from another discipline (i.e., computer engineering—Tangherlini et al. 2020) have also started looking at the narrative aspect of CTs and, more specifically, the Greimassian approach to narratives. Through a quantitative analysis, Tangherlini et al. (2020, p. 33) find that “a conspiracy theory is characterized by a comparatively small number of actants, multiple interconnected domains, and the fragility of the narrative framework graph, which can easily be disconnected into a series of disjoint subgraphs by the deletion of a small number of nodes or relationships.” These conclusions, which complement my own, should spur further studies focused on narratives.

Returning to our discipline, the findings in part revive some of the foundational debates among virtue ethicists interested in business (and business ethicists in general). Among the narratives studied in this research, how many of the authors of these narratives would agree with MacIntyre (1981) on the irrelevance of the current—but still modern—business application of theories of ethics and how far current practices are from their ideal? How many, in fact, have the same position as MacIntyre (1981) but express themselves with their own words and stories through BrCTs? More generally, the findings indirectly raise the question of the possibility for virtue to exist in the business arena. The findings are a troubling echo of what Wicks (1996) says about the separation thesis, that is, the separation between business and ethics, which one is tempted to make but should be rejected (Freeman 1994). Wicks (1996, p. 111) says that.

the separation thesis is especially disturbing because of the insidiousness and pervasiveness of the assumptions that support it and the far-reaching and debilitating effects it has on our work. The emergence of this thesis in the literature has made it especially important for us to look at our literature with a critical eye.

Is history somehow repeating itself here with BrCTs? Business ethicists have debated how, for instance, virtue fits into business (Whetstone 2001), the notion of *character* is sometimes misused (Solomon 2003), CSR “can be equated to the practice of the virtue of mercy” and should be part of the practice of justice (Arjoon 2000, p. 172), and whether business consists of a MacIntyrean practice—one that is based on excellence—or depends too much on effectiveness instead of excellence (Beadle 2008; Moore 2005; Sinnicks 2019b). Given the findings, it seems conspiracists could discard all these academic discussions based on a few statements that they hold to be true. For example, they would say that the business world is not, at present, a network of communities of practice with virtuous agents working toward the common good and that it rarely has been and will not be so as long as (1) the asymmetry of power between citizens (represented by the state) and business continuously increases in favor of business and (2) the MacIntyrean practice is unable to resist the attractive and corruptive external goods, such as money, status, or power, because justice and transparency are not guaranteed—largely because of statement #1. In other words, they would respond to our academic articles by asking us, in return, whether we honestly believe that virtue can thrive in the current neoliberal business environment, which does not even guarantee the safety of many people’s lives.

More generally, the study of narratives offers a possible avenue to further explore mundane conversations and confront them with the ethical world of business that we have socially constructed in academia. This research indirectly underlines how the wide variety of online data—considered here as narratives—can be a remarkable space from which many ethical issues can be addressed and, hopefully, solved. The Internet is a contemporary agora made of legion spaces in which masked people feel safe enough to share their thoughts. By considering online data as the visible outputs of humans’ signifying practices and thoughts, researchers may want to use narratology to reveal concealed meanings, as well as the underlying dynamics and complexities that structure the relationships between individuals, society, and business.

As this research introduces a new research theme in business ethics, I conclude by proposing two paths for future research: One calls for digging into BrCTs and is motivated by the desire to combat them; the other suggests taking advantage of conspiracy narratives to challenge our discipline and concepts such as CSR, notably to avoid falling into ethical blindness and an unwise practice of wisdom.

### Fighting BrCTs

A BrCT is just one more narrative world offered to people. Some enter this narrative world with hindsight and bemusement when they are not ethically distressed, while others use it from time to time to explain a certain lack of understanding about reality, and others, unfortunately, lock themselves into this narrative world and deny any other narrative world. Below, I propose a four-part agenda structured around the aim of the research, which consists of helping institutional and individual agents protect themselves and others from BrCTs.

#### Helping Institutional Agents Protect Themselves

This section of the agenda is dedicated to research projects that study the antecedents and consequences of BrCT belief for institutional agents, especially when they become the target of conspiracists. For instance, are employees who perceive a conspiratorial workplace more likely to act unethically in and outside their work environment? In this scenario, employees could feel either powerful because they are part of the company or cynical about the business world to the point of losing their virtue. While Douglas and Leite (2017) start with an answer by showing that employees imagining a conspiratorial workplace are more likely to leave that workplace, further research focusing specifically on ethical behavior is still needed. Other projects should study the different escape narrative strategies available to institutional agents when they are wrongly attributed the role of the Benefited or Volunteer and evaluate which strategies prevent backfire effects. Additionally, the institutions’ characteristics need to be linked to people’s belief in BrCTs. For example, the institutional agents’ level of CSR involvement or perceived power based on their age, size, nationality, or media spending could influence the belief in BrCTs.

#### Helping Institutional Agents Protect Others

This section of the agenda is dedicated to research projects that study how institutional agents, in a CSR logic, can protect stakeholders from conspiracy narratives. For instance, social media companies should protect not only consumers but also their content moderators, who are supposed to steer consumers away from conspiracy narratives and eventually end up believing in conspiracy theories (Newton 2019). At a time when tech companies such as Google and Facebook are investing millions of dollars to combat fake news and conspiracy theories while the EU is asking them to work harder (Chee 2019), research should focus on how effective these actions are and how to get the most out of technology without forgetting that conspiracy narratives are, above all, moral stories that people need to hear when they feel ethically distressed and potentially suffer from ethical blindness. In this respect, would banning videos, apps, accounts, or websites—as reasonable as it may sound—prevent believers from developing an immoral imagination? Ethical questions also have to be raised: What about freedom of thought and speech, for instance, if institutional actions prevent the population from learning, thinking, and sharing their ideas? Where is the boundary? Finally, one critical research avenue deals with how amateurs and believers of BrCTs can regain trust in business.

#### Helping Individual Agents Protect Themselves

This section of the agenda is interested in research spotting the antecedents and consequences of a belief in BrCTs for individuals. For instance, research in psychology stipulates that a belief in conspiracy theories satisfies epistemic, existential, and social motives (Douglas et al. 2017). To what extent are perceptions of control, agency, and conspiracist behavior related to each other? Does a strong internal locus of control influence the story that people tell themselves and others as well as the related actions they take? How? In addition to the psychological functions of a belief in BrCTs, another stream of research should deal with the more experiential aspect of this belief. According to 2003, there has been a critical spread of CT in the New Age milieu since the 1990s, which has led to reprints of The Protocols of the Elders of Zion. Similarly, Ward and Voas (2011) argue that conspiracy discourses are close to metaphysical ones. As the experiential discourse became a major discursive strategy in esoteric literature (Hammer 2001), one can hypothesize that BrCTs would have not only psychological functions but also experiential ones that are critical for people as they become disenchanted with the world. In this case, one may consider BrCT believers as members of a cultic milieu, that is, a “cultural underground of society”—in opposition to the dominant culture—offering “quasi-scientific beliefs” and convincing its members that all they “ever need to know will eventually be made clear” (Campbell 1972, p. 128–134).

#### Helping Individual Agents Protect Others (and Preventing Them from Harming Others)

This final section of the agenda mainly focuses on the mechanisms that lead individuals to decide to share a BrCT and why some BrCTs spread faster than others, such as the *Plandemic* documentary video that seeks to link Big Pharma, Bill Gates, the WHO, and COVID-19 with each other. It should also answer questions like, “how to react to someone who shares a BrCT online?” While answers can already be found, at least in part, in psychology, this paper has highlighted the power of narratives and their ethical dimension. Therefore, there is a question of whether one should enter the narrative to better counter the theory—and with which strategy. On a related note, what weight do I give the same BrCT when it is shared by an opinion leader, a relative, or an unknown person? Do BrCTs, which are often the subject of mockery among the general public, spread in a similar way as rumors, or is there a different mechanism? Finally, research should also understand and identify factors that potentially undermine unethical behavior caused by a belief in BrCTs. As an example, someone who believes climate change is a conspiracy can keep living as they have always lived or may decide to pollute even more by consuming more because of this belief. In the latter case, how can unethical consumption behavior be limited?

### What Political CSR Can Learn from BrCTs

Political CSR is based on a well-accepted assumption that is also found in BrCTs: the weakening of the state and power’s increasing asymmetry with business. In conspiracy narratives, it is not clear whether governments and, more generally, non-corporate organizations are playing the Volunteer actantial role (i.e., being at the service of the Benefited) or maintaining their original Targeted actantial role. For instance, if lobbying still shows that governments and international institutions could be considered Targeted actors, corruption scandals also show people that institutions could be Volunteer actors. While institutional bodies often remain the last chance for actors to see unethical practices being punished, conspiracy narratives reveal profound suspicions about governments’ role and interests, as well as doubts about their ability to stay on a level playing field with “Big Business.” This is potentially problematic as conspiracy narratives often make non-corporate institutions the dei ex machina that trigger catharsis, that is, the purification of emotions (Aristotle 1962). Catharsis in theater has a normative function as it shows the audience that doing the right thing is always rewarded and those who are guilty are punished. The problem in BrCTs is that non-corporate institutions appear to have too little power compared with the business world, which lacks virtue in the eyes of BrCTs believers and amateurs. Therefore, catharsis cannot be triggered and may potentially increase ethical distress that leads to ethical blindness and belief in BrCTs.

Articles on political CSR emerge and, agreeing that the state’s status is diminishing, advance the idea of increased governance of companies, especially multinationals (MNCs), to compensate for the state’s failings (for reviews and discussion, see Frynas and Stephens 2015; Scherer and Palazzo 2011; Scherer 2018). This possibility must be given careful consideration by researchers since it challenges a long tradition clearly separating politics and society from business (Mäkinen and Kourula 2012). The point here is not to reject the idea of political CSR outright—politics and business have long been intertwined (Acosta and Pérezts 2019; Djelic and Etchanchu 2017), but to be certain why business ethicists think of and latch onto this idea. To continue the discussions and debates on political CSR (Whelan 2012; Scherer 2018), I invite researchers to question political CSR from an even more critical perspective, as some have started to do (Néron 2016; Singer 2018), or in line with the critical perspectives already taken on CSR (Banerjee 2008; Jones 1996). For example, Kourula and Delalieux (2016) discuss political CSR by taking the case of a French children’s clothing retailer that, in the name of ethics, allows dominant groups to manage waves of discontent while strengthening its hegemonic position. While these power relations are studied in Kourula and Delalieux (2016) from a Gramscian perspective, new research should also be based on the work of Foucault (1975, 2004), whose work on power, close to that of Gramsci (1971), has the advantage of not being associated with a political theory (i.e., Marxism in the case of Gramsci).

Foucauldian concepts can remind business ethicists how the birth of an idea does not just simply happen, rationally, as if it were a logical sequel to the world. For instance, Foucault (2004) puts forward the concept of *governmentality* to describe how power is not necessarily associated with force and laws but with the subtle conduct of individuals who, through positive means that make them regulate themselves, lead these same individuals to find it rational to be governed in a particular way. More generally, part of the research projects in business ethics should focus on the less obvious dynamics of neoliberal capitalism, the ideologies that construct the concept of political CSR, as well as the disadvantaged groups that are most in need of CSR but are not heard about often enough. For this purpose, I close the paper by challenging our community with two questions that I consider important and in line with Kallio’s (2007) call to *break taboos* in business ethics: What if political CSR was the product of governmentality and a lack of practical wisdom? And how does the other half live?

#### Political CSR: The Product of Governmentality and a Lack of Practical Wisdom?

Here I raise the possibility that political CSR is merely the result of a neoliberal ideology of which we, researchers, are prisoners, sometimes without even realizing it. One of the lessons of this research into BrCTs is the public’s perception that society is turning into a huge market without virtue; one in which people do not feel safe and are nostalgic for an external force to regulate this market, partly because of an anthropological stance that leads individuals to be pessimistic altruists (i.e., they want to be good to others but have very little trust in these others). In this context of mistrust toward corporations and their motivations, is it wise to develop a concept of political CSR that makes the power relationship between the state or non-corporate institutions (i.e., external forces), on the one hand, and business, on the other hand, more unbalanced? It is from this fundamental question that we must start in order to understand and justify—or not—the political projects of CSR.

Perhaps the beginning of a Foucauldian response would be to think of political CSR, which emerged in the 1980s around the idea of corporate citizenship (Vidaver-Cohen and Altman 2000), as the result of governmentality exercised by neoliberal capitalism, whose current form also became popular around the 1980s. While weakening the state by being its executioner, neoliberal capitalism has managed to make us think it was the best positioned—or the least misplaced—to become a legitimate authority on which the population could rest. This proposal is a continuation of existing criticisms of CSR, namely that CSR is a political project stemming from the neoliberal imagination and whose objective is to protect the interests of the ruling class that holds the capital (Fleming and Jones 2013; Shamir 2008). I would add here that, by taking the place of non-corporate institutions and relying on the voluntary action of economic agents, CSR can have a perverse effect. For example, political decisions on climate change are now taking too long because they are based on voluntary action and the neoliberal idea that it is up to everyone to change their behavior. I believe this approach to CSR throws the very concept of CSR into disarray and is potentially harmful as it strengthens not only conspiracy thinking but also political thoughts, such as eco-authoritarianism, by arguing in favor of undemocratic actions to overcome the weakness of the state and the excessive latitude given to corporations.

I am also fueling this debate with another important point of this research: pessimistic altruism, reflected in the need to have an environment conducive to virtue in order not to fall into the selfishness of the market. Virtue can only be exercised in a favorable environment in which individuals feel safe enough not to appeal to their survival instincts (Aristotle 2009; Hobbes 1651/1996; MacIntyre 1981; Smith 2003). Are we in a position today to affirm that the neoliberal environment in which we find ourselves bears no resemblance at all to the Hobbesian state of nature, to the point of letting economic agents regulate the lives of citizens? Where the state confuses individuals and encourages virtue by protecting them, the market distinguishes between individuals and puts them in competition, thus creating a sense of insecurity that is unfavorable to virtue. In the same vein, Foucault (2004) suggests that the neoliberal ideology is conquering and spreading throughout the entire social fabric to the point where the economy is no longer at the service of man; on the contrary, our entire society becomes subject to the economy, with the market as the mechanism allowing the state to function. In extending this thought, let us imagine that the current crises in our economies and the weakening of the state, which are giving way to ever more competition between precarious individuals, are a sign of the contemporary state of nature. In such a scenario, are we sure that our decision to rely on companies to govern us is not, as with the belief in BrCTs, the result of a lack of Aristotelian practical wisdom?

#### How Does the Other Half Live?

At a time when business research is debating political CSR and how it can—or should not—be implemented, there is one overlooked yet significant stakeholder: the citizen. What do citizens expect from CSR and, more specifically, from political CSR? While much CSR research focuses on consumers, employees, or even imagines stakeholder democracy (Crane et al. 2004), business ethics may sometimes be forgetting to *spend time* with the citizens who have a voice and the power to choose for themselves and their community. Citizens are not just consumers or employees; they exist beyond the economy. By installing companies in the heart of the village, political CSR must be a public debate open to all citizens and even more so to vulnerable citizens. Like Jacob Riis (1890/1996), who was interested in the other 50%^7^ of New York’s 19th-century population whom we never see and whose opinion we never ask, I encourage research among vulnerable populations who represent a large part of our societies to ask them what they think of political CSR while their precarious living conditions have often resulted from the market. For example, we know that globalization is increasingly perceived as a risk rather than an opportunity in Germany (Bidder 2020). The same is true in France, where 60% of the population has a bad opinion of globalization, particularly because of free trade and the hegemony of multinationals (OpinionWay 2018). I also urge business ethicists to interview all those citizens who would not even understand what we are talking about when we talk to them about political CSR and to ask them what they think of the business world. As an example, 67% of the French population does not know what CSR is, and about two-thirds of the 33% who have heard the term are unable to say exactly what it is (IFOP 2019). As suggested by Silver (2015) through a contractualist analysis of the 2010 Citizens United v. Federal Election Commission case decided by the US Supreme Court, citizens’ vulnerability can easily be used to benefit companies. This is also our role as researchers in business ethics: to ensure that all actors involved in the process of politicizing business understand what is at stake for them, can make their voices heard, and can influence this process. If this is not done, we potentially and indirectly contribute to making today’s citizens tomorrow’s subjects at the service of the economy.

## Abbreviations

BrCT: Business-related conspiracy theory
CSR: Corporate social responsibility
CT: Conspiracy theory

## Footnotes

1 Source: www.who.int/emergencies/ten-threats-to-global-health-in-2019

2 In the sense of MacIntyre (1981/2011, p. 218), that is “any coherent and complex form of socially established cooperative human activity through which goods internal to that form of activity are realized in the course of trying to achieve those standards of excellence which are appropriate to, and partially definitive of, that form of activity, with the result that human powers to achieve excellence, and human conceptions of the ends and goods involved, are systematically extended.”.

3 Many YouTube videos have since been deleted, as parent company Google has been the subject of several investigations regarding the content of videos available on YouTube.

4 The data comes from SimilarWeb and concerns the period between December 2018 and May 2019.

5 More information about the fake rice conspiracy can be found here: https://observers.france24.com/en/20170614-investigation-%E2%80%9Cplastic-rice%E2%80%9D-part-2-origins-african-internet-hoax or here: https://medium.com/@matthijsbijl/chinas-post-factual-rice-problem-in-africa-2c37e3a8237a

6 For instance, Apple states on its website that “This is and always has been about our customers. We feel strongly that if we were to do what the government has asked of us — to create a backdoor to our products — not only is it unlawful, but it puts the vast majority of good and law abiding citizens, who rely on iPhone to protect their most personal and important data, at risk.” See also Tim Cook’s 2016 message to customers here: https://www.apple.com/customer-letter/

7 In his introduction, Jacob Riis (1890/1996, p. 59) writes that, “Long ago it was said that ‘one half of the world does not know how the other half lives’.”
